# Multilevel Influences on Providers' Delivery of Contraceptive Services: A Qualitative Thematic Analysis

**DOI:** 10.1089/whr.2021.0128

**Published:** 2022-05-09

**Authors:** Abbey K. Mann, Amal Khoury, Paezha McCartt, Michael G. Smith, Nathan Hale, Kate Beatty, Leigh Johnson

**Affiliations:** ^1^ Department of Family Medicine, Quillen College of Medicine, and College of Public Health, East Tennessee State University, Johnson City, Tennessee, USA.; ^2^ Department of Health Services Management and Policy, College of Public Health, East Tennessee State University, Johnson City, Tennessee, USA.

**Keywords:** contraceptive behavior, qualitative research, primary care

## Abstract

**Introduction:**

Access to a full range of contraceptive services is essential for quality health care. Contraceptive provision practices of primary care providers play an important role in patients' decision-making about their reproductive health care. Understanding the multilevel factors influencing contraceptive care delivery in primary care settings is critical for advancing quality care. This study offers an in-depth examination of influences on providers' delivery of contraceptive services across multiple primary care specialties and practice settings to identify issues and strategies to improve care.

**Materials and Methods:**

Twenty-four in-depth face-to-face interviews were conducted in 2017 with primary care providers, including family physicians, gynecologists, pediatricians, and nurse practitioners from academic settings, private practices, and health centers. Interviews were transcribed and analyzed thematically.

**Results:**

Providers described a complex set of influences on their provision of contraception across multiple ecological contexts. Seven major themes emerged from the qualitative analysis, including six types of influence on provision of contraception: organizational, individual provider-related, structural and policy, individual patient-related, community, and the lack of influences or barriers. Providers also discussed the sources they access for information about evidence-based contraception counseling.

**Conclusions:**

A diverse set of providers described a complex system in which multiple concentric ecological contexts both positively and negatively influence the ways in which they provide contraceptive services to their patients. To close the gaps in contraceptive service delivery, it is important to recognize that both barriers and facilitators to patient-centered contraceptive counseling exist simultaneously across multiple ecological contexts.

## Introduction

Reproductive health care, including access to the full range of contraceptive services, is fundamental to patients' health and well-being. Modern contraception is safe and effective, allowing reproductive life planning and preventing unintended pregnancy.^1–4^ Primary care providers play a key role in contraceptive counseling and provision.^5–7^ Provider practices and quality of care are a major driver of contraceptive use in the population and ultimately of reproductive health outcomes.^8,9^

Researchers have noted gaps in the delivery of evidence-based contraceptive counseling and the full range of contraceptive methods.^10–17^ Studies identify individual provider characteristics associated with contraceptive provision, including provider type/specialty,^15,18^ provider training (knowledge, skills), and attitudes/beliefs,^11,12,16–19^ but much of the literature focuses on specific specialties such as emergency medicine or pediatrics.^19,20^

Little is known about influences on providers' contraceptive practices beyond their individual characteristics, such as policy, community, and organizational factors, and whether these influences vary or not across primary care specialties. In addition, much of the literature has focused on specific contraceptive methods such as emergency contraception or long-acting reversible contraception (LARC),^19,21^ and there is limited research about influences on contraceptive counseling.

This study adds to the literature in a meaningful way by offering an in-depth examination of multilevel influences on providers' contraceptive counseling and provision, including policy, community, and organizational factors. This study is novel in assessing both positive and negative influences across multiple primary care specialties (family physicians, pediatricians, obstetricians/gynecologists [obs/gyns], nurse practitioners) and practice settings (private practices, publicly funded clinics, academic settings) to identify the most salient issues and strategies to improve care. Understanding the multilevel factors influencing contraceptive care in primary care settings is critical for informing health policy and clinical practice and advancing accessible high-quality care.

In addition, our study focuses on providers in South Carolina (SC), a population that has not been studied in this context before. Examining contraceptive provision in SC is important given that the state has one of the lowest rates of “wanted-then-or-sooner” pregnancies,^22^ and more than one in four reproductive aged women in SC do not use any contraception.^23^ Providers and patients in SC face a challenging sociodemographic landspace. SC experiences a higher poverty rate than the national average and 25 of the state's 46 counties are rural.^24^

It is estimated that more than 300,000 women in need in SC live in contraceptive deserts—counties where the number of health centers offering the full range of methods is not enough to meet the needs of the county's number of women eligible for publicly funded contraception.^25^ The legislative and political environment in the state also presents barriers to contraceptive provision and access. SC did not expand Medicaid under the Affordable Care Act,^26^ and nearly 16% of reproductive aged women in SC remain uninsured.^25^

Sex education in SC schools is limited by restrictive requirements,^27^ and the state does not perform favorably on reprodutive health and reprodutive rights policy.^28^ As such, examining factors impacting contraceptive delivery in SC is important for advancing patient-centered care and reproductive autonomy in this historically underserved state while contributing to the national discussion around accessible, quality family planning. In addition, to the extent that the health policies and organizational climates that affect contraceptive access and use are similar across the U.S. South,^22,28^ this study findings could have implications for advocacy and programmatic efforts in other southern states.

## Materials and Methods

### Design and eligibility

This study is a cross-sectional qualitative study in which we conducted 24 in-depth, face-to-face interviews in 2017. Interviewees represented diverse primary care specialties, practice settings, and geographic regions of SC. To be eligible for this study, providers had to meet all three eligibility criteria: (1) office-based family physician, ob/gyn, pediatrician providing adolescent care, or nurse practitioner (women's health nurse practitioner or family nurse practitioner); (2) major professional activity in primary care (*i.e.*, provider spent most of the time in outpatient patient care, not in administration, teaching, research, or other activity); and (3) provided contraceptive and/or HIV/sexually transmitted infection services.

We focused on these medical specialties because they provide the majority of reproductive health care to patients. Study procedures were approved by the East Tennessee State University Institutional Review Board.

### Sample identification and recruitment

Eligible providers were identified using purposive and snowball sampling. A “maximum variation” purposive sampling approach maximized heterogeneity of participants across four criteria (medical specialty, practice setting, geographic location across the four regions of the state, and rurality) and captured a wide range of provider perspectives related to contraceptive attitudes and practices. Recruitment strategies included (1) nominations for interviewees from women's health leaders at public and private agencies in SC, (2) recruiting providers from the SC Rural Health Association conference, and (3) asking interviewees for recommendations of potential participants.

Study staff sent invitation letters to a total of 39 providers. Providers who did not respond within 1 week received a reminder email and telephone follow-up to clarify the purpose of the study and answer questions. A total of 24 providers accepted the invitation (62% participation rate). Interviews were scheduled at a time and place convenient to the providers, typically at their practice location.

### Data collection tools

We collected data using a semistructured discussion guide and a brief demographic survey. The guide, consisting of a series of open-ended questions with probes, was informed by an extensive literature review and collected data about contraceptive practices and sources of influence on contraceptive care delivery. The demographic sheet collected data about provider's age, gender, race/ethnicity, clinical degree, medical specialty, years in practice, practice setting, zip code, board certification, faculty status, time allocated for patient care, and patient volume. The interview guide and demographic survey were piloted with a small sample of providers, including physicians and nurse practitioners, revised and finalized.

### Data collection

We conducted interviews in the fall of 2017. Two members of the research team traveled to the providers' offices, with the project leader consenting the providers and conducting the interviews and the research assistant taking field notes. Interviews typically lasted 45–60 minutes each, and we sent providers a $100 remuneration for their time. Data saturation was achieved with 24 participants, and data collection was concluded in December 2017. We audio recorded and transcribed interviews, and we appended field notes appended to the transcripts.

### Variables of interest

Analysis for this study focused on seven interview questions (see Appendix A1 for a complete list of questions) designed to elicit information from providers regarding factors that influence the ways in which they counsel about and prescribe contraception. These questions included an explicit focus on information that helps providers stay up-to-date on contraceptive prescribing guidelines, how similar or different a provider's practice is compared with peers, organization, policy and community influences, and patients' unmet needs.

### Analysis

Using a thematic analysis approach,^29^ the first and third authors, read through the transcribed data and identified an initial list of codes through open coding. The authors then applied the initial codes to a section of the data independently, after which we collaboratively organized the codes into themes, revised the codes, and applied the codes to additional data. We repeated this process until all data were coded and we were in agreement about the application of codes and themes to the whole data set. The second author, was given the codebook and a set of responses to which she applied these codes without knowledge of how the data were coded by the first two authors.

This process resulted in 80% agreement between codes applied by the first two authors and the additional coder. Minor revisions were made to the codebook and application of codes, and the resulting agreement regarding coding of the data was 100%. Analysis was performed with NVivo 1.0.^30^

## Results

### Description of the sample

We conducted interviews with a diverse sample of 24 providers, including 8 nurse practitioners, 5 pediatricians, 4 family physicians, and 7 ob/gyns (see Table 1 for participant characteristics). In terms of practice settings, 9 providers were in private medical practice, 7 in academic settings (university or hospital clinic), and 8 practiced at health centers. Of the 24 providers, 9 practiced in rural areas. Collectively, participating providers saw diverse clients including adolescents and adults with varied racial and ethnic backgrounds across multiple income levels, insurance status (privately insured, publicly insured, uninsured), and geographic locations (rural and urban/suburban).

**Table 1.** Participant and Clinic Characteristics (*n* = 24 Interviews)

Participant characteristics	No. of participants (%)
Gender	
Male	5 (21)
Female	19 (79)
Age	
Range	30–74
Mean	47.48
Standard deviation	11.15
Race/ethnicity	
White	18 (75)
Black/African American	4 (17)
Mixed race	2 (8)
Provider specialty	
Family physician	4 (17)
Obstetrician/gynecologist	7 (29)
Pediatrician	5 (21)
Nurse practitioner	8 (33)
Years in practice	
Less than 5 years	3 (12.5)
5–10 years	6 (25)
10–15 years	3 (12.5)
15–20 years	3 (12.5)
20 or more years	9 (37.5)

Clinic characteristics	No. of clinics (%)
Geography	
Rural	9 (38)
Urban	15 (62)
Region of the state	
Upstate	5 (21)
Midlands	6 (25)
Pee Dee	7 (29)
Low country	6 (25)
Primary practice setting^a^	
Academic (university or hospital clinic)	7 (29)
Private office practice	9 (38)
Health center^b^	8 (33)

^a^ Of the 24 interviews, 5 were at rural health clinics, including 4 private offices and 1 health center.

^b^ Of the 8 health centers, 6 were federally qualified health centers 1 was a college health center, and 1 was a hospital-affiliated center.

### Findings

Authors identified seven major themes that emerged from the analysis of providers' responses: organizational, provider-related, structural and policy, patient-related, community, absence of barriers or influences, and sources of information about contraception.

#### Organizational

Of the 24 providers interviewed, 21 mentioned organization-related barriers and facilitators to contraceptive provision. Specifically, about half of the providers mentioned difficulty of getting appointments and lack of available providers as organization-related barriers to contraceptive care. For example, one provider expressed a desire for patients to get same-day visits at their clinic, noting that patients might want birth control 1 day but could change their mind if they have to wait 2 weeks for an appointment.

More same day visits. If somebody calls and says, “I want birth control” they want birth control’, they want it then. In two weeks they may change their mind.—Family nurse practitioner, college campus

Another provider talked about access, in terms of clinic location, walk-in visits, and same-day service provision, as an organizational facilitator of contraceptive care.

… you can walk in and get those services without having to go see someone, potentially go home, go again to get whatever it is contraception that you want to get. …That model where you don't have to have an appointment and you can get the contraceptive services that you need that day, I think is ideal… if the contraceptives are free but you can't get there and you can't get to it, then it doesn't matter.—OBGYN, private practice, urban area

Providers also mentioned challenges related to the need for clinic profitability, with some noting that they wish they could offer care for free or for less than what they currently charge to increase access for patients. A related concern raised by some providers was the expense of having LARC devices in stock and the delay in care that having to order devices on a case-by-case basis creates.

A few providers discussed supportive organizational policies. For example, a couple of providers specifically mentioned that their organization's policies are supportive of them offering contraception to pediatric patients. One provider mentioned the negative influence of some organizational policies, including a previous policy in the organization against providing contraception or supporting provider training for contraceptive practices. Another provider, a pediatrician in an academic setting, mentioned that his or her organization is “not restrictive” and “fairly forward-thinking” when it comes to providing contraception to adolescents.

#### Provider-related

Most (20) participants brought up provider-related influences on contraceptive provision. Participants indicated that provider experience, training, and educational background influence the ways in which they practice.

I think that we as providers may not have as much knowledge, primary care may be behind the times in what we're throwing out there, and what we are offering.—Family Nurse Practitioner, health center, rural area

Participants also mentioned that they or other providers have individual preferences or varying comfort with procedures or practices that guide the way they provide contraception, including some participants, most of whom were not pediatricians, who mentioned that pediatricians might be less comfortable than other primary care providers addressing reproductive health issues with patients. Some participants expressed their and others' discomfort in prescribing intrauterine devices (IUDs) to teens.

…probably half of the pediatricians didn't even want to mess with birth control. They talked to their patients right in front of their parents. “You're not having sex right?” Yes, because you're going to wait until you're married and all this stuff…I get to meet pediatricians from all over the country. I find that almost none of them screen their patients for HIV. …The Nexplanon or IUD, I don't know a single pediatrician that does that. Now listen, medicine is a different story. A general pediatrician, I've not met one that does that.—Pediatrician, health center

Some other participants cited other providers' religious beliefs as influencing whether or how they provide contraception for patients.

We do have quite a few in this particular practice that are adverse [to providing contraception] for their own religious beliefs. They tend to refer them to some of us who are comfortable with doing contraceptive care. I'd say that probably there's a greater group than I would have expected to see that are not willing to prescribe or use any kind of contraception for their patients—Family nurse practictioner, health center

Notably, most participants talked about provider preference or experiences, about half talked about provider training and education as a barrier or facilitator, and a quarter mentioned that provider beliefs play a role. All three subcategories were mentioned by providers from each specialty represented in the sample.

#### Structural and policy

Nineteen providers identified issues beyond the organization at the structural or policy level that influenced their care, primarily insurance coverage, billing and reimbursement issues related to insurance, specific policies/laws, cost of contraceptives to clinics, and transportation. Half of the participants mentioned that uninsurance or underinsurance poses a barrier to their ability to provide care that best meets the contraceptive needs of their patients and talked about challenges related to billing and reimbursement for the services they provide.

I do have several patients that have decided just to go with the OCPs because that's what they can afford. Because they can't afford $600, $700 for the placement of an IUD or $800 for Nexplanon.—OBGYN, private practice, rural area

Relatedly, several providers talked about the barriers that the general cost of getting contraceptive care poses to their patients and their inability to provide lower fees or participate in government pricing.

… the health departments get 340B pricing, it's government pricing so they can get their pills and stuff cheap. I know when I was there we paid like a penny a cycle for pills. But I can't get that even though we're a state supported agency… I don't want to charge for my services to be able to. I like not charging and them being able to come see me without paying a fee.—Nurse, college campus

Beyond financial barriers, several providers mentioned it can be difficult for patients to get transportation to their clinic, and while there are efforts in some communities to improve access through transportation, these efforts do not help enough people.

Transportation is an issue because a lot of patients come from rural areas; some of them, up to 50 miles away. I'm always impressed, when they tell me they do that. I'm like, “Wow. Thanks. Nice to know” It makes access a little bit of an issue for them.—Pediatrician, health center

Some providers also mentioned efforts to increase access *via* transportation that had been successful, such as one who described a program that takes girls from school to the clinic and back.

We also have the transportation that helps bring the patients in and out… If there's any kids that need these types of things, that parents have agreed…they can transport them during school hours…They'll be seen and then brought back to school so they can finish the rest of the day,…They'll transport them to wherever they need to go to a specialized.—Family nurse practitioner, private practice

A few providers commented on specific laws related to contraceptive care provision, such as the law that prevents them from providing contraceptive care in school settings.

…we can't do school based clinics and provide contraception in schools. That's illegal in our state, but having clinics that are in locations that are more accessible to teens would be really helpful.—OB/Gyn, academic setting

While some providers only discussed their frustration with certain laws and policies, others described ways in which they had worked around such issues to connect with patients despite these barriers.

#### Patient-related

Many providers (17) brought up patient-specific issues that influenced their provision of contraception. Provider talked about patients' lack of knowledge or misconceptions about reproductive health in general and contraception options specifically, as well as limited awareness of the availability of family planning clinics as barriers to their ability to prescribe contraception. Potential patients might not know what their options are or where they can go to get care.

… I think there's a lot of people out there who aren't aware that they can get some of those services through a place like [clinic name], through a federally qualified health center… So I think the awareness of the availability is probably the biggest.—Nurse, health center, suburban area

Several providers talked about the importance of listening to patients' preferences about their contraception options, and several providers also mentioned the role that parents sometimes play in the process of getting contraception, particularly for pediatric patients, but also for older teenagers including those starting college. Parents' role can be supportive of contraception or not, with some parents encouraging their sexually active teens to use contraception, including long-acting methods, and accompanying them to their appointments, while other parents seeming to ignore or deny sexual activity.

A few providers noted that patient compliance influences the choices providers make about providing contraception. Providers discussed why they may be more likely to recommend LARCs for patients whom they suspect will not comply with user-dependent methods, but even there, patients may not show up to their LARC appointment.

Then there's the patient compliance issue. You set aside 30 or 45 minutes for a contraceptive counseling session, a postpartum visit and the patient is alleged to want an IUD. You've got it, or you've requisitioned it from the pharmacy. The patient doesn't show up. Unfortunately, that happens much more frequently than we need for it to.—OB/gyn, academic setting

#### Community

Half of the providers interviewed (12) mentioned community-situated influences on their ability to provide contraception. These influences can be negative or positive.

Several providers mentioned predominant religious beliefs and stigma in the community as negative influences on contraception provision, such that conservative religious beliefs and negative attitudes about contraception could pose as barriers to care.

I think it's still a little bit of a taboo to talk about contraception. For a long time I think the only form of contraception was abstinence, so to even suggest that there was something to do, to use to prevent pregnancy other than abstinence was just unheard of. … That still we shouldn't be talking about those things and you shouldn't need to worry about contraception because you're not going to have sex until you're married ….- Family Physician, academic setting, suburban area

On the contrary, a few participants shared examples of community acceptance and support of their practices, making it easier to connect patients with contraception, and highlighted the positive role of community reaching organizations. A few providers described generally good relationships with the surrounding communities and community-based groups.

…the community at large is largely receptive to the provision of contraceptive services.—OB/Gyn, private practice

#### Sources of information

Providers noted a range of different sources of information about contraceptive care. Most providers pointed to more than one source of information. The most frequent responses were other providers, conferences, peer-reviewed literature, and formal guidelines, such as American College of Obstetricians and Gynecologists, or online resources such as UpToDate. Notably, five providers said that they do not follow a specific set of guidelines or that they do not find them helpful. For example, one ob/gyn in a private practice responded “I don't think I follow any formal guidelines.”

A couple of providers stated that providers have freedom to practice based on their preferences, rather than following specific guidelines, and one mentioned that they felt guidelines were not nuanced enough to be helpful in their contraceptive provision pratices.

## Discussion

This study used in-depth interviews to closely examine multiple levels of influence on provider counseling about and provision of contraception. Our findings illustrate similarities in influences across providers with multiple specialties. Providers discussed structural/policy, organizational, community, patient, and personal influences on their contraception provision, in addition to formal sources of information. The majority of providers cited community factors and the majority of providers cited all other factors , illustrating how common and widespread these influences are on contraceptive delivery. Responses add depth to our understanding of the complex network of interrelated factors affecting contraception counseling and provision.

In contrast with much of the previous research, which examined influences on clinical decision-making,^31–35^ the current findings extend our knowledge by identifying contextual influences on care, in addition to organizational and individual factors, across multiple provider specialties and practice settings.

Across organization types, providers identified organization-related factors that echo previous findings, such as the ability to make same-day appointments, and availability of long-acting contraception.^36,37^ They also discussed organization-related issues not previously noted in the literature, such as the need for clinic profitability and the role of organizational leadership and policies. This may be because these issues were beyond the scope of previous research, but their identification here indicates that a focus on the role of the organization is a complex one that is recognized by a range of providers.

Much of the previous literature on provider-related influences on contraception provision focused on specific specialties and on particular methods of contraception.^19–21^

In this study, providers from a mix of specialties noted provider-related factors—specifically their preferences or previous experiences with contraception provision, their training or education, and their beliefs—play an important role in how they counsel about and prescribe contraception. These factors were not all described as barriers, as some providers noted that their high degree of knowledge and experience facilitates care. Nevertheless, lack of experience, confidence, and comfort in counseling about and providing the full range of contraceptive methods emerged as a common theme. This highlights the need for training of providers while in medical school and residency and also as a part of continuing medical education.

Previous researchers have identified providers' perceptions of cost as a barrier to contraception,^19,38^ although the structural barriers, such as limited insurance reimbursement or the high cost to clinics of obtaining IUDs, raised in this study have received less attention.

Although previous work has focused on the cost and lack of insurance as barriers,^39,40^ there is less evidence on providers' perspectives on these significant barriers.^36^ Moreover, providers in this study recognized the role of specific policy issues, lack of patients' access to transportation, and legal impediments to contraception provision. These findings indicate that providers recognize that the factors influencing their contraception provision are complex and work simultaneously across multiple ecological conctexts They also recognize that some of these factors extend beyond the walls of the clinic and may be largely beyond their control.

Some providers recognized patient preferences as important factors to consider during the counseling process to facilitate shared decision-making and patient-centered care. Other providers talked about the limited knowledge of their patients and, as highlighted in the results, a possibility for patients to “change their mind” about wanting contraception, as negatively affecting their preferences and awareness of their contraceptive options. This highlights the need for community outreach and education programs to raise patients' awareness of their options and for provider training that emphasizes a patient-centered approach to contraception counseling.

There may be an opportunity for providers to further acknowledge their role and responsibility in counseling and informing their patients, as well as an opportunity for training programs to prepare providers to better address patients' misperceptions about contraception. Providers in this study also dicussed the role of patients' parents in the process of contraception counseling. Parents' own beliefs and experiences may influence those of their children. This warrants additional study, particularly when adolescent patients are the population of interest.

Little of the previous literature focuses on community-related factors that influence contraceptive provision. Only one study mentions the role of religion but does so in the context of individual patients.^36^

Participants in this study noted that religion, community acceptance or support, and stigma from community members play a role in their provision of contraception. These findings point to the need for additional close examination of the ways in which religious communities, community support, and stigma affect providers' perceptions and practices, particularly given the omnipresence of evangelical religious beliefs and practices in this region.^41^ In addition, these findings point to an opportunity to promote models of collaboration with faith-based organizations to help raise community support for contraceptive care.

Interestingly, there was little consensus among providers as to where they get their information about contraceptive guidelines and evidence-based practices. Providers mentioned a range of sources of information including some that may not be highly reliable.

This study has some limitations including that its intent is not to serve as a representation of all contraception providers, but rather to closely examine the influences on this sample in this context. The study also only included providers who prescribe contraception. We may be underestimating or missing some barriers or influences that would have been voiced by providers who do not provide contraception. In addition, given that more providers in the sample were white than non-white, female than male, and more practiced in urban areas than in rural areas, we may have picked up on a wider range of themes related to the experiences of those providers than we would have if we had included more providers of color, more male providers, and more providers in rural areas.

Providers' recognition of the mutlilayered system of influences on their contraceptive practices is an indication that single-level approaches, such as increased training alone, may not be seen by providers as having much potential for long-term change. Instead, multilevel initiatives and approaches to improving access to contraceptive care will be more acceptable to providers, who are highly aware of these complex barriers. Providers identified factors that are potentially modifiable by policymakers and organizational leaders, such as more robust reimbursement and changing policies that prevent reproductive health education schools. Recognizing and addressing influences across multiple contexts simultaneously will facilitate patient care.

## Conclusion

Across practice settings and specialties, providers in this study painted a picture of a complex web of intersecting influences on their contraception counseling and provision that included factors at multiple levels including structural, organizational, community, patient, and personal. Some of these nonclinical influences are positive and appropriate in reaching the best decision for an individual patient, whereas other influences may contribute to a disparity in contraceptive practices and to unequal treatment of patients across population groups, practice settings, and communities.

## Abbreviations Used

IUDs: intrauterine devices
LARC: long-acting reversible contraception
obs/gyns: obstetricians/gynecologists
SC: South Carolina

## Appendix

### Appendix A1. Interview Guide

1. How do you remain up-to-date on current recommendations for contraceptive prescribing?
2. a. What guidelines do you follow to determine if the patient is eligible for one contraceptive method over another?
2. In your opinion, how useful are clinical guidelines in contraceptive care delivery?
4. a. Which sets of guidelines do you find the most helpful?
5. b. What are the barriers you face in utilizing those guidelines?
3. To what extent is your approach to contraceptive delivery similar or different from the approach of your colleagues/peers?
4. In what ways does your organizational culture of policies impact your delivery of contraceptive services to your patients?
5. How would you say your community and/or social environment impact your delivery of contraceptive services to your patients?
6. Do you see an unmet need for contraceptive services in your community?
7. Is there anything else about this topic that you feel is important to discuss?
